# Corporeal rehabilitation to manage acute stress in critically ill patients

**DOI:** 10.1186/s13613-022-01019-3

**Published:** 2022-06-10

**Authors:** Irma Bourgeon-Ghittori, Maryline Couette, Sylvie Marini, Rachida Ouedraogo, Aline Alves, Keyvan Razazi, Damien Carras, Ann-Cecile Pallud, Nancy Kentish-Barnes, Armand Mekontso Dessap

**Affiliations:** 1 Groupe de recherche CARMAS, Univ Paris Est Créteil, 94010 Créteil, France; 2 INSERM, IMRB, Univ Paris Est Créteil, 94010 Créteil, France; 3 DMU SAPHIRE, AP-HP, Hôpitaux Universitaires Henri-Mondor, 94010 Créteil, France; 4 Service de Médecine Intensive Réanimation, Hôpitaux Universitaires Henri-Mondor, AP-HP, 1 Rue Gustavec Eiffel, 94010 Créteil, France; 5 Groupe de Recherche Famiréa, Service de Médecine Intensive Réanimation, CHU Saint-Louis, AP-HP, 94010 Paris, France

**Keywords:** Intensive care, Stress, Corporeal, Rehabilitation

## Abstract

**Background:**

Intensive care unit (ICU) patients often endure discomfort and distress brought about by their medical environment and the subjective experience of their stay. Distress, pain, and loss of control are important predictors of future neuropsychiatric disorders. Depression, anxiety, and post-traumatic stress are common after discharge. We aimed at mitigating acute stress and discomfort via a novel intervention based on body image rehabilitation and rehabilitation of senses performed following a holistic approach guided by positive communication (corporeal rehabilitation care, CRC).

**Results:**

We conducted a prospective observational study on 297 consecutively enrolled patients participating in at least one CRC session. Benefits of CRC were assessed on both subjective analogical scales of stress, pain, and well-being criteria, and objective clinical measures of dyspnea, respiratory rate, and systolic arterial pressure, just after CRC and long after (a median of 72 min later) to estimate its remote effect. Results showed that CRC had a positive effect on all overt measures of distress (acute stress, pain, discomfort) just after CRC and remotely. This beneficial effect was also observed on dyspnea and respiratory rate. Results also showed that best CRC responders had higher baseline values of stress and heart rate and lower baseline values of well-being score, indicating that the care targeted the population most at risk of developing psychological sequelae. Interestingly, a positive CRC response was associated with a better survival even after adjustment for physiologic severity, indicating a potential to identify patients prompt to better respond to other therapeutics and/or rehabilitation.

**Conclusion:**

This study demonstrated the feasibility of an innovative holistic patient-centered care approach and its short-term positive effects on critical parameters that are considered risk factors for post-intensive care syndrome. Further studies are warranted to study long-term benefits for patients, and overall benefits for relatives as well as ICU staff.

## Introduction

Intensive care unit (ICU) workers manage critically ill patients at high risk of death because of vital organ failure using invasive treatments which expose patients to a wide range of physical injuries, stressful events, pain, and discomfort [1–4]. All exert additional physical burden on patients already exhausted by the disease. The technical facet of critical care and the physical deterioration of patients may urge healthcare providers to distance themselves emotionally at the expense of losing empathy towards their patients. Most often, invasive techniques are deployed on the “body-as-object” to save the life of the “body-as-subject”, which carry the risk of dehumanization.

However, patients expect healthcare providers not only to have technical skills, but also to show empathetic behavior. They need to be listened to and to share their emotional and cognitive experience. This exchange should help clinicians understand where their patients come from as well as their uniqueness. Patients expect kindness and compassion in response to their vulnerability and suffering. They wish not to be reduced to their illness [5], but rather be placed at the center of care. Patient-centered care seems to be the legitimate alternative to the disease-centered model. A new approach that focuses on each person’s specific health needs and desired health outcomes in order to optimize health care decision-making. Some patient-centered care approaches have been proved effective in reducing discomfort [6], promoting relaxation [7–9], and mitigating physiological indicators of discomfort or stress like blood pressure and heart rate [10].

Corporeal rehabilitation care (CRC) is a complex intervention with a holistic approach including rehabilitation of patient’s image (esthetic care to restore physical integrity of patient) and rehabilitation of senses (e.g., touch, hearing, and smell, via several sensorial inputs), all guided by positive communication. This multisensory approach is crucial since bodily self-consciousness encompasses integration of all sensory information via a huge cerebral network [11–15]. Brain structures are drastically affected during ICU stay by stress hormones and administered psychotropic drugs, and both alter consciousness [16, 17].

We hypothesized that CRC may mitigate acute stress and its potential consequences in critically ill patients. The aim of this prospective observational study was dual. First, to assess the effect of CRC on psychological (stress, well-being) and functional (pain, dyspnea, and cardiorespiratory system) markers. Second, to scrutinize the characteristics of CRC responders, as compared with non-responders.

## Method

### Patients and characteristics

Patients admitted to the medical ICU of *Henri-Mondor* University Hospital between October 2018 and August 2021 were included in this prospective study if they fulfilled the following criteria: aged more than 18 years, hospitalized for at least 24 h and having a Richmond Agitation-Sedation Scale (RASS) between − 1 and + 2 [18]. The ICU staff (nurses and physicians) decided in collegiality, during their pluri-professional meetings, which patients could benefit from CRC based on the subjective or objective identification of any significant psychological stressor. The objective criteria were obvious verbalization of stress, pain or discomfort by the patient, their loved ones or caregivers. The subjective criteria were body or facial expressions clearly evoking pain, stress and/or discomfort. Patients were not included if they did not understand written and/or oral French language, if refused to participate, if have deafness, dementia (MMSE score under 20), psychosis, extensive burns, Lyell disease, or moribund status. The protocol was approved by the Institutional Review Board as a component of standard care; accordingly, patient’s consent was requested as per French law and both written and oral information about the protocols were given to the patient. The patient’s healthcare professional and the socio-esthetician invited the patient to participate in this study.

### Corporeal rehabilitation care

**Fig. 1:**
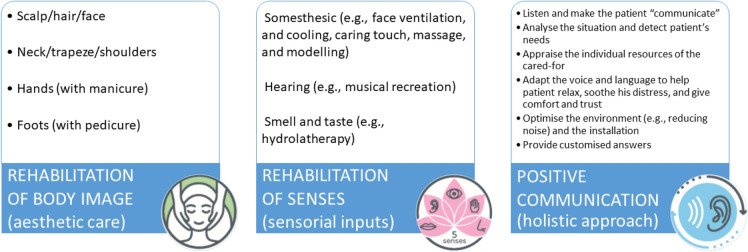
Corporeal rehabilitation care

CRC was delivered by a CODES-certified (*Cours d’Esthétique à Option humanitaire et Sociale*) socio-esthetician, a healthcare professional distinct from the nurse or assistant-nurse of the patient [19]. A CRC session of 30 min, on average, was carried out in daytime, and consisted of two components (body image rehabilitation and rehabilitation of senses) performed following a holistic approach guided by positive communication (Fig. 1). Body image rehabilitation included esthetic care where cosmetics were applied to the scalp/hair, neck/trapeze/shoulders, face, and other parts of the body like manicure for hands and pedicure for feet, in order to restore physical integrity of patient. Rehabilitation of senses used several sensorial inputs and involved somesthesic (face ventilation, cooling, caring touch, massage, and modeling), hearing (musical recreation), and smell and taste (hydrolatherapy). Positive communication comprised the following good professional practices: ability to listen and to make the patient “communicate”; capacity to analyze the situation and detect patient’s needs; capacity to appraise the individual resources of the cared-for; capacity to adapt the voice and language in order to help patient relax, soothe his distress, and give comfort and trust, all by optimizing the environment (reducing noise, optimizing installation); and finally, the capacity to provide customized answers.

### Outcomes

The primary outcome was the change in psychological variables (stress and well-being) immediately after CRC (direct evaluation) and long after (remote evaluation). Secondary outcomes included: (i) the change in functional parameters, like pain, dyspnea, respiratory rate, and blood pressure; (ii) the factors associated with a positive response to CRC and its outcome.

Three analog scales running from 0 to 10 were used to assess pain, acute stress, and well-being, with higher values representing pejorative status, as follows: no pain = 0, unbearable pain = 10; no stress = 0, unbearable stress = 10; excellent well-being (no discomfort) = 10, minimal well-being (unbearable discomfort) = 0. Analog scales have been tested before to assess pain and stress in adults [20]. To assess dyspnea, we used a five-item scale (heart rate, neck muscle indrawing during inspiration, abdominal paradox, facial expression of fear, and supplemental oxygen) derived from the original intensive care respiratory distress observation scale (IC-RDOS) [21, 22]. Each parameter is scored from 0 to 2 points and the final score is the sum of all points. Scale scores range from 0 signifying no distress to 10 signifying the most severe distress. This derived version has been proved useful to assess dyspnea in ICU patients unable to communicate and/or use visual analog scale [23]. All questionnaires were recorded by an investigator not involved in patient care.

In order to characterize CRC responders, as compared with non-responders, we assessed the following variables: patients’ characteristics, past medical history, long-term treatments, reasons for admission, severity score at ICU admission, organ function and support (hemodynamic, respiratory, and neurological) at inclusion, complications during ICU stay, duration of CRC, and type of care delivered. A positive response to CRC reflected a significant improvement in at least one of the three: stress, well-being, or pain (with a decrement of at least 2 points on stress or pain scale after CRC or an increase of at least 2 points on well-being scale after CRC). Sessions with a baseline stress/pain score of less than 2 and those with a baseline well-being score of more than 8 could not be assessed for CRC response. For a given patient, we only considered the first CRC to be assessed for the response.

At direct evaluation, we also assessed CRC appreciation by the patient (usefulness, satisfaction, and desire to renew care). Finally, ICU length of stay, and vital status at day 28 were assessed.

### Statistical tests

Data were analyzed using SPSS Base 24.0 statistical software package (SPSS Inc, Chicago, IL). Continuous data were expressed as median [interquartile range]. Paired continuous variables were compared using nonparametric analysis of variance (Friedman test) and Wilcoxon paired test (with Benjamini–Hochberg correction for multiple testing when appropriate). Independent continuous variables were compared by Mann–Whitney test. Categorical variables, expressed as numbers and percentages, were evaluated using Chi-square test or Fisher exact test. To evaluate independent factors associated with a positive response to CRC, significant bivariate risk factors (using the above mentioned tests) were examined using univariate and multivariable backward stepwise logistic regression analysis. Coefficients were computed by the method of maximum likelihood. The calibrations of models were assessed by Hosmer–Lemeshow goodness-of-fit statistic (good fit was defined as *p* value > 0.05) [19] and discrimination was assessed by the area under the receiver operating characteristics curve (ROC-AUC, where 1 indicates perfect discrimination and 0.5 indicates the effects of chance alone). Two-tailed *p* values of less than 0.05 were considered significant. A Cox model was used to assess the effect of a positive response to CRC on day-28 mortality while adjusting for patient’s physiologic severity and for the Simplified Acute Physiology Score (SAPS) 2.

## Results

### Patients characteristics

Among 3401 patients admitted in our ICU during the study period, we enrolled 297 patients who underwent 323 CRC sessions (three patients had three sessions, 20 patients had two sessions, and 274 patients had a single CRC session). Median age of the 297 patients was 59 [37–71] years old, and 130 were males (44%). Median SAPS 2 score was 32 [18–47] and 78 (26%) patients were on invasive mechanical ventilation at time of inclusion.

### CRC sessions

The CRC session was provided at a median of 3 [2–8] days after ICU admission, and lasted a median of 25 [20–30] minutes. CRC consisted in applying esthetic care in the form of cosmetics, massage, and modeling on the face (*n* = 229, 71%), neck, trapeze, and shoulders (*n* = 46, 14%), scalp (*n* = 42, 13%), and other body parts (*n* = 55, 17%), in addition to hands manicure (*n* = 71, 22%), and feet pedicure (*n* = 60, 19%). During CRC, all but two patients were sedative-free, while 72 (22%) were on intermittent morphine. RASS score in the 24 h preceding CRC was 0 [0–0].

### Effects of CRC

**Fig. 2:**
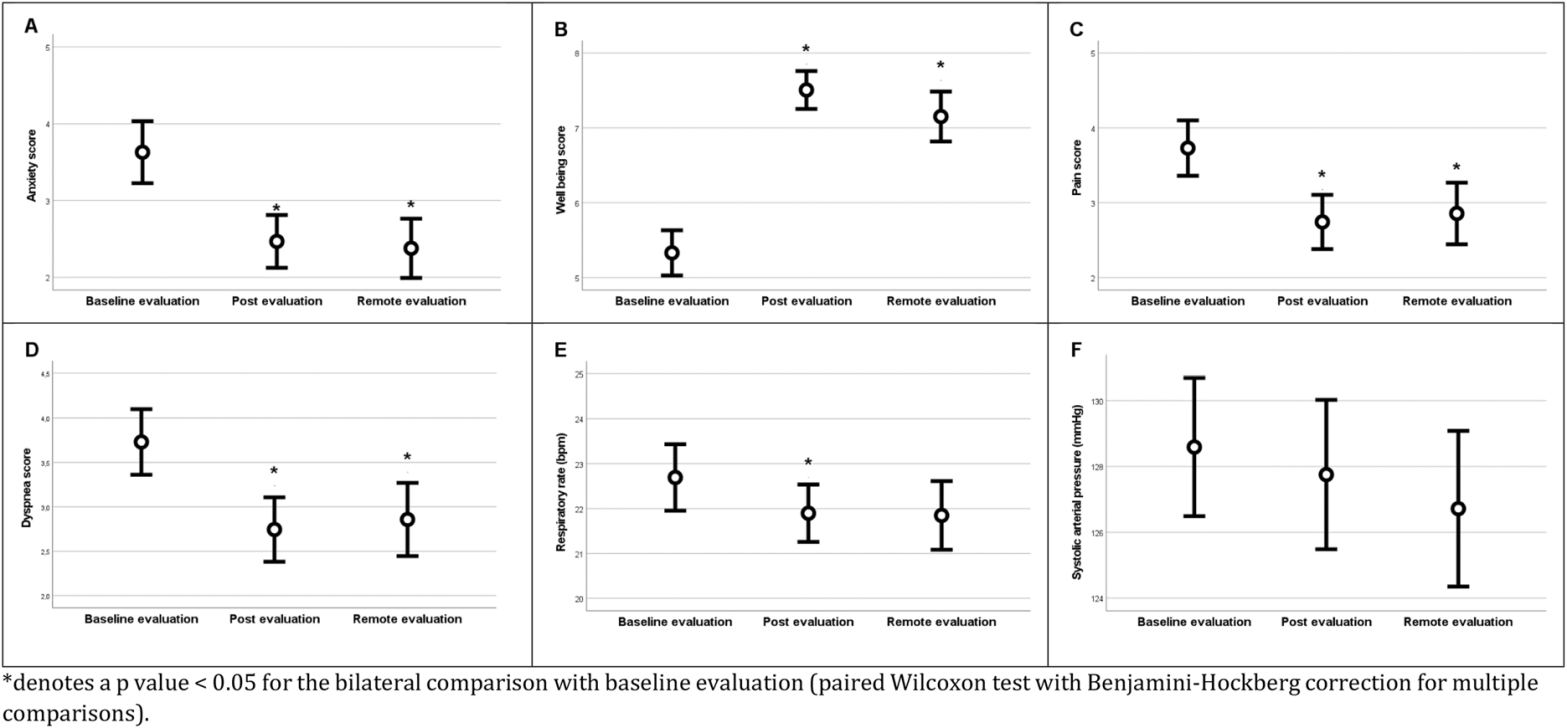
Effect of corporeal rehabilitation care on psychological [stress (**A**), well-being (**B**)], and functional [pain (**C**), dyspnea (**D**), respiratory rate (**E**) and systolic arterial pressure (**F**)] variables

The median time between baseline assessment and CRC start, between CRC end and direct evaluation, and between CRC end and remote evaluation was 15 [5–30], 8 [3–15] and 72 [57–94] minutes, respectively. The patients found CRC very useful (score of 10 [8–10]) and were very satisfied (score of 10 [8–10]). We were able to evaluate the psychological effect of CRC in almost all sessions (*n* = 313, 97%). CRC sessions significantly improved the feeling of well-being and significantly decreased stress and pain, both at direct evaluation and at remote evaluation. Moreover, the sessions significantly decreased the respiratory rate and dyspnea score, both at direct evaluation and at remote evaluation (Table 1, Fig. 2).

**Table 1:** Effect of corporeal rehabilitation care on psychological and functional variables

	Baseline evaluation	Direct evaluation	Remote evaluation	*p* value^#^
Pain score	4 [0–6]	2 [0–5]*	2 [0–5]*	< 0.001
Stress score	3 [0–7]	0 [0–5]*	0 [0–5]*	< 0.001
Well-being score	5 [4–7]	8 [6–10]*	8 [5–10]*	< 0.001
Dyspnea score	2.4 [1.1–2.9]	2.2 [1.0–2.5]*	2.2 [1.0–2.5]*	0.011
Respiratory rate	22 [17–27]	21 [18–25]*	21 [17–26]	0.004
SAP (mmHg)	128 [115–141]	128 [113–141]	125 [113–137]	0.035

*SAP* systolic arterial pressure

^#^Friedman test

*Denotes a *p*-value < 0.05 for the bilateral comparison with baseline evaluation (paired Wilcoxon test with Benjamini–Hochberg correction for multiple comparisons)

### CRC responders

Among the 297 included patients, we were able to assess the response to CRC at direct evaluation in 287, of whom 188 (66%) showed response (responders), and 99 (35%) did not (non-responders). The positive response rate varied according to the evaluated items: 74/256 (29%) for pain, 85/256 (33%) for stress, and 138/254 (48%) for well-being. Tables 2 and 3 display patients’ characteristics according to their response to CRC. At direct evaluation, CRC responders experienced a greater decrease in stress, pain, and dyspnea, along with a greater improvement in well-being, as compared with non-responders. The responders found CRC more useful and had higher satisfaction than the non-responders. At remote evaluation, CRC responders reported a greater decrease in stress and pain along with a greater improvement in well-being, but with similar effect on dyspnea, as compared with non-responders.

**Table 2:** Characteristic of critically ill patients, overall and depending on their response to corporeal rehabilitation care

Parameter	*N*	All patients (*n* = 287)	Non-responders (*n* = 99)	Responders (*n* = 188)	*p* value
Patients’ characteristics					
Female gender	287	164 (57%)	44 (44%)	120 (64%)	0.002
Age (years)	287	59 [37–70]	63 [48–72]	55 [36–70]	0.047
Body mass index (kg/m^2^)	257	25 [21–29]	24 [21–28]	24 [21–29]	0.904
Past medical history					
Respiratory disease	287	101 (35%)	34 (34%)	67 (36%)	0.827
Cardiac disease	287	135 (47%)	55 (56%)	80 (43%)	0.036
Neurological disease	287	35 (12%)	10 (10%)	25 (13%)	0.431
Cirrhosis	287	7 (2%)	3 (3%)	4 (2%)	0.696
Cancer	287	44 (15%)	19 (19%)	25 (13%)	0.188
Blood cancers	287	26 (9%)	14 (14%)	12 (6%)	0.030
Sickle cell disease	287	65 (23%)	16 (16%)	49 (26%)	0.057
Chronic kidney failure	287	12 (4%)	4 (4%)	8 (4%)	> 0.99
Psychiatric disease	287	30 (11%)	12 (12%)	18 (10%)	0.503
Alcohol consumption	287	26 (9%)	8 (8%)	18 (10%)	0.675
Drug addiction	287	8 (3%)	1 (1%)	7 (4%)	0.270
Long term treatments					
Benzodiazepine	287	27 (9%)	12 (12%)	15 (8%)	0.253
Antidepressive drug	287	32 (11%)	11 (11%)	21 (11%)	0.988
Neuroleptic	287	18 (6%)	8 (8%)	10 (5%)	0.359
Between ICU admission and inclusion					
SAPS 2 score	281	32 [18–47]	36 [22–48]	32 [15–47]	0.125
Medical admission	287	262 (91%)	87 (88%)	175 (93%)	0.137
Infection	287	174 (61%)	67 (68%)	107 (57%)	0.076
Septic shock	287	62 (22%)	24 (24%)	38 (20%)	0.430
Orotracheal intubation	287	73 (25%)	23 (23%)	50 (27%)	0.534
Tracheostomy	287	11 (4%)	4 (4%)	7 (4%)	> 0.99
ARDS	287	29 (10%)	13 (13%)	16 (9%)	0.217
24 h preceding inclusion					
Maximal SAP (mmHg)	287	143 [129–157]	140 [130–156]	145 [129–158]	0.283
Minimal SAP (mmHg)	287	81 [72–90]	80 [73–89]	81 [72–90]	0.810
Maximal RASS score	287	0 [0–0]	0 [0–0]	0 [0–0]	0.980
Minimal RASS score	287	0 [0–0]	0 [0–0]	0 [0–0]	0.426
Maximal temperature (°C)	287	37 [37–38]	37 [37–38]	37 [37–38]	0.999
Maximal heart rate (bpm)	287	99 [88–114]	99 [84–109]	99 [89–116]	0.202
Minimal heart rate (bpm)	287	80 [69–91]	75 [64–87]	82 [71–93]	0.004
Shock	287	16 (6%)	7 (7%)	9 (5%)	0.423
Noradrenaline infusion^a^	287	12 (4%)	4 (4%)	8 (4%)	> 0.99
Dobutamine^a^	287	5 (2%)	4 (4%)	1 (1%)	0.050
Antihypertensive drug	287	37 (13%)	11 (11%)	26 (14%)	0.514
Continuous sedation or analgesia	287	16 (6%)	2 (2%)	14 (7%)	0.057
Propofol	287	5 (2%)	0 (0%)	5 (3%)	0.168
Fentanyl	287	2 (1%)	0 (0%)	2 (1%)	0.547
Sufentanyl	287	12 (4%)	2 (2%)	10 (5%)	0.229
Morphine	287	73 (25%)	22 (22%)	51 (27%)	0.364
Morphine cumulative dose (mg)	33	57 [6–221]	35 [5–113]	120 [6–324]	0.240
Neuroleptic	287	2 (1%)	0 (0%)	2 (1%)	0.547

*CRC* corporeal rehabilitation care, *ICU* intensive care unit, *SAPS* Simplified Acute Physiologic Score, *ARDS* Acute Respiratory Distress Syndrome, *SAP* systolic arterial pressure, *DAP* diastolic arterial pressure, *MAP* mean arterial pressure, *RASS* Richmond Agitation-Sedation Scale

^a^Dose did not change during CRC

**Table 3:** Baseline evaluation, direct and remote assessment in critically ill patients, overall and depending on their response to corporeal rehabilitation care

Parameter	*N*	All patients (*n* = 287)	Non-responders (*n* = 99)	Responders (*n* = 188)	*p* value
*Baseline evaluation*					
Pre-inclusion time (days)*	287	3 [2–7]	3 [2–7]	3 [2–7]	0.613
Days of IMV before inclusion	72	8 [5–22]	7 [4–23]	9 [5–20]	0.582
Hemodynamics					
Heart rate (bpm)	287	90 [79–104]	90 [78–102]	90 [80–104]	0.612
SAP (mmHg)	287	127 [115–141]	127 [115–141]	128 [114–141]	0.750
DAP (mmHg)	287	70 [62–80]	68 [60–79]	71 [63–82]	0.048
MAP (mmHg)	287	92 [82–101]	92 [82–98]	92 [82–102]	0.504
Comfort					
Pain score	263	4 [0–6]	3 [0–6]	4 [0–7]	0.108
Stress score	263	4 [0–7]	0 [0–5]	5 [0–7]	< 0.001
Dyspnea score	112	2 [1–3]	2 [0–3]	2 [1–3]	0.690
Well-being score	259	5 [4–7]	6 [5–8]	5 [3–6]	< 0.001
Discomfort	287	45 (16%)	15 (15%)	30 (16%)	0.858
Neurological function					
Continuous sedation or analgesia	287	10 (4%)	1 (1%)	9 (5%)	0.173
Midazolam	287	1 (0%)	0 (0%)	1 (1%)	> 0.99
Sufentanyl	287	9 (3%)	1 (1%)	8 (4%)	0.171
Neuroleptic	287	2 (1%)	0 (0%)	2 (1%)	0.547
Morphine	287	64 (22%)	18 (18%)	46 (25%)	0.224
Physical disability	287	20 (7%)	5 (5%)	15 (8%)	0.354
Ventilation					
IMV	286	25 (9%)	9 (9%)	16 (9%)	0.848
Assist-control mode	287	2 (1%)	2 (2%)	0 (0%)	0.118
Tidal volume (mL)	30	517 [420–591]	455 [394–254]	541 [478–642]	0.081
Tidal volume (mL/kg IPW)	28	8 [7–10]	8 [6–9]	9 [7–10]	0.248
End-tidal CO_2_ mmHg	25	33 [29–38]	33 [28–38]	31 [28–37]	0.755
FiO_2_ (%)	39	35 [30–45]	35 [30–48]	35 [30–40]	0.739
Oxygen flow (L/min)	217	0 [0–2]	0 [0–2]	0 [0–2]	0.937
SpO_2_ (%)	287	97 [95–99]	97 [95–99]	97 [95–99]	0.586
Respiratory rate (bpm)	287	22 [18–27]	22 [18–28]	22 [18–27]	0.635
Intercostal retraction	287	5 (2%)	2 (2%)	3 (2%)	> 0.99
Paradoxical breathing	287	8 (3%)	2 (2%)	6 (3%)	0.719
Last available blood gases					
pH	235	7.43 [7.39–7.47]	7.43 [7.40–7.47]	7.43 [7.38–7.46]	0.213
PaO_2_/FiO_2_ ratio (mmHg)	234	333 [238–417]	333 [217–416]	339 [250–418]	0.664
PaCO_2_ (mmHg)	234	38 [32–44]	36 [33–44]	38 [32–44]	0.741
Bicarbonates (mmol/L)	234	26 [23–29]	26 [23–29]	26 [23–29]	0.771
Lactates (mmol/L)	234	1 [1–2]	1 [1–2]	1 [1–2]	0.941
SaO_2_ (%)	235	96 [94–98]	96 [94–98]	96 [94–98]	0.745
*Corporeal rehabilitation care (CRC)*					
Pre-evaluation time^#^ (min)	186	15 [5–30]	10 [4–27]	18 [7–31]	0.122
Face and forehead massage	287	204 (71%)	70 (71%)	134 (71%)	0.919
Manicure	287	61 (21%)	19 (19%)	42 (22%)	0.535
Pedicure	287	50 (17%)	19 (19%)	31 (17%)	0.566
Scalp massage	287	37 (13%)	13 (13%)	24 (13%)	0.930
Other interventions	287	51 (18%)	17 (17%)	34 (18%)	0.847
Duration of CRC (min)	287	25 [20–30]	25 [20–30]	25 [25–30]	0.016
*Direct evaluation*					
Time from CRC^$^ (min)	174	8 [3–15]	10 [2–17]	7 [4–15]	0.646
Hemodynamics					
Heart rate (bpm)	286	89 [78–101]	84 [72–102]	91 [80–100]	0.105
SAP (mmHg)	286	128 [112–140]	129 [111–144]	127 [113–139]	0.661
DAP (mmHg)	286	71 [62–79]	69 [61–78]	72 [62–81]	0.186
MAP (mmHg)	286	91 [80–102]	93 [80–101]	91 [80–102]	0.742
Comfort					
Pain score	258	2 [0–5]	3 [0–6]	2 [0–5]	0.223
Stress score	258	2 [0–5]	1 [0–5]	2 [0–5]	0.555
Dyspnea score	112	2 [1–2]	2 [1–2]	2 [1–2]	0.347
Well-being score	257	8 [6–10]	7 [5–8]	8 [6–10]	< 0.001
Discomfort	286	7 (2%)	3 (3%)	4 (2%)	0.694
Change in comfort^£^					
Δ1 Pain score	256	0 [− 2 to 0]	0 [0–0]	− 1 [− 3 to 0]	< 0.001
Δ1 Stress score	256	0 [− 2 to 0]	0 [0–0]	− 1 [− 3 to 0]	< 0.001
Δ1 Dyspnea score	112	0 [− 2 to 0]	0 [− 1 to 0]	0 [− 2 to 0]	0.870
Δ1 Well-being score	254	2 [0–4]	0 [0–1]	3 [1–5]	< 0.001
Ventilation					
Respiratory rate (bpm)	286	21 [18–25]	21 [17–26]	21 [18–25]	0.820
Intercostal retraction	286	4 (1%)	2 (2%)	2 (1%)	0.609
Paradoxical breathing	286	2 (1%)	1 (1%)	1 (1%)	> 0.99
Patient appreciation of the CRC session					
Usefulness	256	10 [8–10]	8 [7–10]	10 [8–10]	< 0.001
Satisfaction	256	10 [8–10]	9 [8–10]	10 [8–10]	0.002
Patient’s wish to renew care	260	249 (96%)	68 (94%)	181 (96%)	0.503
*Remote assessment*					
Time from CRC^$$^ (min)	131	71 [57–93]	63 [50–94]	72 [60–94]	0.165
Hemodynamics					
Heart rate (bpm)	224	89 [79–100]	86 [76–100]	90 [80–100]	0.379
SAP (mmHg)	224	125 [112–138]	123 [109–134]	126 [113–139]	0.114
DAP (mmHg)	224	71 [60–80]	67 [57–78]	72 [61–82]	0.005
MAP (mmHg)	224	90 [79–100]	86 [77–97]	91 [81–102]	0.035
Discomfort	223	10 (5%)	2 (3%)	8 (5%)	0.728
Comfort					
Δ2 Pain sore	204	2 [0–5]	0 [0–5]	2 [0–5]	0.752
Δ2 Stress score	203	0 (0–5]	0 (0–5]	0 (0–5]	0.705
Δ2 Dyspnea score	111	2 [1–2]	2 [1–3]	2 [1–2]	0.723
Δ2 Well-being score	203	8 [6–10]	7 [5–9]	8 [6–10]	0.043
Change in comfort^£^					
Δ2 Pain score	202	0 [− 2 to 0]	0 [0–0]	0 [− 2 to 0]	0.012
Δ2 Stress score	202	0 [− 2 to 0]	0 [0–0]	− 1 [− 3 to 0]	< 0.001
Δ2 Dyspnea score	111	0 [− 1 to 0]	0 [0–0]	0 [− 1 to 0]	0.673
Δ2 Well-being score	202	1 [0–4]	0 [0–1]	2 [0–4]	< 0.001
Ventilation					
Respiratory rate (bpm)	224	21 [17–26]	20 [17–25]	22 [18–26]	0.431
Intercostal retraction	223	2 (1%)	1 (1%)	1 (1%)	0.530
Paradoxical breathing	223	2 (1%)	2 (3%)	0 (0%)	0.98
*Outcome*					
ICU stay (days)	287	6 [4–12]	6 [4–12]	7 [4–14]	0.528
Death at day 28	287	22 (8%)	15 (15%)	7 (4%)	0.001

Δ1: absolute difference between the value at direct evaluation and the value at baseline; Δ2: absolute difference between the value at remote evaluation and the value at baseline

*IMV* invasive mechanical ventilation, *IPW* ideal predicted weight, *CO*_*2*_ carbon dioxide, *FiO*_*2*_ inspired oxygen fraction, *SpO*_*2*_ oxygen transcutaneous saturation

*Days between ICU admission and inclusion

^#^Time between baseline assessment and CRC start in minutes

^$^Time between CRC end and direct intervention assessment in minutes

^$$^Time between CRC end and remote evaluation in minutes

^£^Change in score as compared with baseline

A positive response to CRC was less reported in men, older age, and patients with past medical history of cardiac disease or blood cancers. At baseline, higher values of heart rate, diastolic arterial pressure and stress score and lower values of well-being score were all associated with greater likelihood of positive response. The multivariable analysis showed that the baseline factors independently associated with a positive CRC response were absence of blood cancers, higher heart rate, higher stress score and lower well-being score (Table 4).

**Table 4:** Factors associated with response to corporeal rehabilitation care in critically ill patients

Variable	Odds ratio [95% confidence interval] by logistic regression	
	Univariate	Multivariable
Demographics and past medical history		
Female gender	2.21 (1.34–3.62), *p* = 0.002	I/NR
Age, per year	0.99 (0.97–1.00), *p* = 0.047	I/NR
Cardiac disease	0.59 (0.36–0.97), *p* = 0.037	I/NR
Blood cancers	0.41 (0.18–0.93), *p* = 0.034	0.25 (0.09–0.70), *p* = 0.008
Before CRC		
Minimal heart rate*, bpm	1.02 (1.01–1.04), *p* = 0.008	1.03 (1.01–1.05), *p* = 0.005
Diastolic arterial pressure, mmHg	1.02 (1.00–1.04), *p* = 0.045	I/NR
Stress score	1.18 (1.08–1.28), *p* < 0.001	1.14 (1.04–1.25), *p* < 0.006
Well-being score	0.77 (0.68–0.87), *p* < 0.001	0.80 (0.70–0.91), *p* = 0.001

^*^Measured in the preceding 24 h; I/NR, included, but not retained in the final model; the multivariable model showed a good calibration as assessed by Hosmer and Lemeshow goodness-of-fit test [*χ*^2^ (4 *df*) = 5.31, *p* = 0.72] and a fair discrimination as assessed by the receiver operating characteristics curve [area under the curve of 0.76 (0.70–0.83), *p* < 0.0001]

**Fig. 3:**
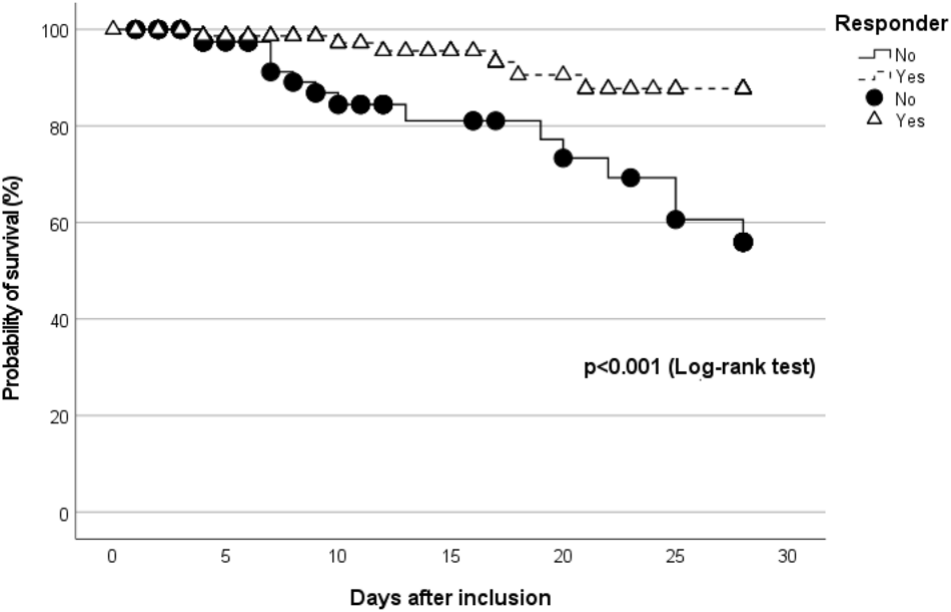
Probability of survival in critically ill patients with or without a positive response to corporeal rehabilitation care

Finally, day-28 mortality was lower in responders as compared with non-responders (Table 3, Fig. 3) and this association persisted after adjustment for SAPS 2 score [odds ratio (95% confidence interval) of Cox model: 0.27 (0.11–0.67), *p* = 0.005].

## Discussion

To the best of our knowledge, we herein report the first experience of CRC in critically ill patients with the following findings: (i) excellent feasibility of this care and significant improvement of most psychological and functional parameters after CRC, with an immediate and remote effect on stress, well-being, pain, and dyspnea; (ii) a positive CRC response is associated with absence of blood cancers, higher heart rate and stress score and lower well-being score before CRC; (iii) a positive CRC response is also associated with a lower day-28 mortality, even after adjustment for patient’s physiologic severity.

### CRC

Overall, patients judged the CRC sessions useful and satisfactory (median of 10 on the 10-point analog scale). This result, along with the positive effects on psychological and functional parameters, encourage us to further develop this innovate approach in the context of critical care. The type of care offered during the session (face and forehead massage, manicure, pedicure, neck and shoulder massage, scalp massage, hydrolatherapy, music, etc.) varied from one patient to another, but did not affect the response. This was probably because each session was adapted to treated patient’s desire which highlights the role of engaging the patients in their care (i.e., patient-centered care). In this way, the patient becomes an agent again and is no longer seen as a passive body dissociated from will and personality. Tailoring CRC to patient’s preferences, in spite of their limited capacity of expression, makes the patient re-embody and affirm their intimate self as a human being in front of themselves as well as the others (nursing staff and/or family). Regaining control of own health and body is crucial as it acts on the agency dimension, expressing one’s will, that in turn impacts all the other dimensions of humanization as stated by Todres et al. [24]. Moreover, patient-centered care enhances ICU healthcare professionals’ compassionate feelings and gives them higher satisfaction of their work [25]. Behavioral science considers humanization of healthcare as an approach that enables to consider the person as a complete and complex being. Analyzing the interactions between healthcare professionals and patients makes it possible to extend the complexity of lived situations to the eight dimensions of humanization/dehumanization: (1) insiderness/objectification; (2) agency/passivity; (3) uniqueness/homogenization; (4) togetherness/isolation; (5) sense-making/loss of meaning; (6) personal journey/loss of journey; (7) sense of place/dislocation; (8) embodiment/reductionist view of the body [24]. ICU is an ultra-technical environment where these eight humanization-guaranteeing dimensions are the most disturbed.

### Response to CRC

The overall response to CRC was favorable in the majority of patients. Higher heart rate, higher stress score and lower well-being score at baseline were associated with a positive response to CRC. Tachycardia and variations in heart rate are well-known biological markers of stress. Two recent meta-analyses highlighted the link between heart rate variability and activity of cerebral areas involved in stressful event processing like amygdala and ventro-medial prefrontal cortex [26, 27]. High heart rate measured during acute traumatic event is significantly associated with the subsequent development of post-traumatic symptoms [28]. A large amount of literature demonstrates the influence of body-centered interventions on psychological illnesses, notably stress disorders and depression [29]. In the polyvagal theory developed by Stephen Porges [30, 31], the vagus nerve is directly connected to the body viscera and their functions (measured by respiratory sinus arrhythmia), thus acts as an indicator of attention, emotions and their self-regulation. It has been shown that massage stimulates the vagus nerve, and as a result increases parasympathetic function and reduces symptoms associated with dysregulation of nervous autonomous system like tachycardia and stress [29].

On the contrary, patients with a past medical history of blood cancers were less prone to show a positive CRC response. The psychological distress and fear of recurrence of blood cancers-associated ailments could explain this lack of response. A recent literature review on survivors of blood malignancies reported that patients suffered from psychological distress even during remissions. Authors describe the so-called Damocles syndrome [32], where patients are free of malignancy but not of fear of recurrence and manifest despair vision of the future expressed by a feeling of uncertainties about life [33]. Patients with a past history of blood cancers could present a pessimistic trait that makes them less sensitive to any type of therapy because they are constantly afraid of disease recurrence and ineffectiveness of care.

The duration of CRC was statistically longer in responders as compared with non-responders, though the medians were similar (25 min); we cannot exclude that the observed positive response encouraged the prolongation of the session in responders.

### Clinical implications

Intensive care induces physical and psychological alterations that may dehumanize the patient and change their self-image. Acute intervention to prevent those disabling psychological symptoms are of particular interest as they may prevent long-term consequences of ICU stay. ICU survivors may develop the well-described post-intensive care syndrome [34, 35]. The latter encompasses physiological sequelae, cognitive and psychiatric disorders like depression, stress, and post-traumatic stress disorder with a high prevalence long after ICU discharge (around 20%) [36]. A recent meta-analysis reported that one third of ICU patients suffer durably from stress 1 year after discharge [37]. In a meta-analysis conducted by Davydow et al. [38] most studies reported a high prevalence of depression in ICU survivors during the year following discharge (around 28% on depression questionnaire and around 33% on clinician interview). Depression, stress, and post-traumatic stress disorder have a clear impact on quality of life; they are known to induce physical complications running from heart disease to inflammatory diseases [39–44]. Acute intervention to mitigate ICU-induced stress and discomfort is crucial to avoid physical health alteration directly related to psychological distress. Our results showed that CRC has a significant effect on these markers of poor prognosis in alignment with previous studies findings as depicted in a recent systematic review on the beneficial effects of massage interventions on ICU patients’ outcomes [45].

### Outcome

Death at day 28 was lower in responders as compared with non-responders, even after adjustment for patient’s physiologic severity. A positive CRC response, irrespective of the patient’s physiologic severity, could identify individuals who are sensitive and compliant with other types of therapeutics (including other rehabilitation programs), and potentially result in a better survival. Patients with a positive response may have an optimistic rather than pessimistic trait. A recent meta-analysis depicted an association between optimistic trait and absence of cardiovascular events and all-cause mortality [46]. Another meta-analysis conducted by Rasmussen et al. [47] highlighted that optimistic trait was a significant predictor of physical health. However, our preliminary results must be taken with caution and further studies are warranted to scrutinize the role of optimistic or pessimistic trait in CRC, its effect and outcome. In fact, other cofounding variables may exist that were not assessed, and later events or treatments, which occurred after the first CRC session, may have influenced the outcome of patients.

### Strengths and limitations

The strengths of our study lie in its large sample size and detailed assessment of psychological and functional variables at direct evaluation and remote evaluation. Our study has some limitations. First, the remote evaluations were relatively close to the end of CRC session (72 min), which precludes the assessment of late effects. In addition, few patients had repeated sessions because of the limited availability of the socio-esthetician.Second, we did not have a control group, and the level of stress and pain was quite low at baseline in the entire cohort, using the 10-point analog scale; however, our analysis showed that a higher level of baseline stress was associated with CRC responsiveness. Third, the type of CRC interventions varied from one patient to another and this may be a limiting factor for external validity. Nonetheless, tailoring the intervention to the patients’ desire in order to engage them in their care is a cornerstone of this approach, since it allows patients to reincorporate their body by expressing their will and their uniqueness as human beings. Fourth, despite being large, the number of subjects included in our study only represents a small proportion of patients admitted to the ICU during the study period. This raises the question of the feasibility and the generalization of this approach in routine care. Fifth, data concerning pain, stress, dyspnea and well-being were lacking for some patients. Eventually, it would have been interesting to estimate the impact of this care on patient’s long-term psychological status, on the healthcare professionals, and on patient’s family.

## Conclusion

In conclusion, CRC proved useful in mitigating acute stress, pain, and discomfort in critically ill patients. The care was well received and well evaluated by the patients, especially those with a greater baseline stress. Responders had a better outcome than non-responders, even after adjustment for patient’s physiologic severity. Further studies are needed to assess the long-term benefits of repeated CRC sessions, and to estimate its impact on the psychological distress of healthcare professionals and that of the family or loved ones.

## Data Availability

Upon reasonable request.
